# Moralischer Stress bei Medizinstudierenden und ärztlichen Berufseinsteigenden: Forschungsdesiderate im Rahmen der COVID-19-Pandemie

**DOI:** 10.1007/s00103-020-03244-2

**Published:** 2020-11-12

**Authors:** Katja Kühlmeyer, Eva Kuhn, Kathrin Knochel, Hanna Hildesheim, Victoria Dorothea Witt, Orsolya Friedrich, Annette Rogge

**Affiliations:** 1 Institut für Ethik, Geschichte und Theorie der Medizin, Ludwig-Maximilians-Universität (LMU), Lessingstr. 2, 80336 München, Deutschland; 2 Philosophisches Seminar, Lehrstuhl für Praktische Philosophie, Christian-Albrechts-Universität zu Kiel (CAU), Kiel, Deutschland; 3 Institut für Geschichte und Ethik der Medizin, Klinikum rechts der Isar der Technischen Universität München (TUM), München, Deutschland; 4 Kinderpalliativzentrum, Klinikum der Ludwig-Maximilians-Universität München (LMU Klinikum), München, Deutschland; 5 Institut für Experimentelle Medizin, Medizinethik., Christian-Albrechts-Universität zu Kiel (CAU), Kiel, Deutschland; 6 Segeberger Kliniken, Neurologisches Zentrum, Bad Segeberg, Deutschland; 7 Fakultät für Kultur- und Sozialwissenschaften, Institut für Philosophie, Juniorprofessur für Medizinethik, FernUniversität in Hagen, Hagen, Deutschland

**Keywords:** Medizinstudium, Psychische Gesundheit, Ethik, Klinische Ethik, COVID-19, Moralischer Stress, Medical school, Mental health, Ethics, Clinical ethics, COVID-19, Moral distress

## Abstract

**Hintergrund:**

Die COVID-19-Pandemie stellt Menschen, die in der medizinischen Versorgung arbeiten, vor besondere Herausforderungen. Ein Teil der Medizinstudierenden und ärztlichen Berufseinsteigenden, die in dieser Zeit in Einrichtungen der Gesundheitsversorgung ihre Mitarbeit beginnen, wird mit außergewöhnlichen moralischen Herausforderungen konfrontiert. Einige verfügen noch nicht über ausreichend Bewältigungsmöglichkeiten, um adäquat mit diesen Herausforderungen umzugehen. Dies kann zu sogenanntem moralischen Stress (MoS; Englisch: „moral distress“, MoD) führen. Dauerhafte oder intensive Belastung durch MoS kann gravierende Folgen haben. Geeignete Unterstützungsangebote haben das Potenzial, den Umgang mit MoS zu verbessern.

**Ziel:**

Der Beitrag hat das Ziel, einen Überblick über den Stand der Forschung zu MoS von Medizinstudierenden und ärztlichen Berufseinsteigenden zu geben, um Lehrende mit Aus- und Weiterbildungsverantwortung und Ärzt*innen in Leitungspositionen für die Problematik zu sensibilisieren.

**Hauptteil:**

In diesem Beitrag werden das wissenschaftliche Konzept MoS, bekannte Auslöser sowie Präventions- und Interventionsmöglichkeiten vorgestellt. Dazu wird das Thema Bezug nehmend auf die Veränderungen in der Patientenversorgung im Kontext der COVID-19-Pandemie analysiert und es werden Forschungsdesiderate aufgezeigt.

**Fazit:**

Der Beitrag verdeutlicht die Notwendigkeit eines deutschsprachigen, interdisziplinären Diskurses über MoS bei Medizinstudierenden und Berufseinsteigenden.

## Abstract (en)

**Background:**

The COVID-19 pandemic poses particular challenges for people working in the medical sector. Some of the medical students and young medical professionals who are starting their work in healthcare facilities during this time are confronted with extraordinary moral challenges. A portion of them does not yet have sufficient coping skills to adequately deal with these challenges. This can lead to so-called moral distress (MoD). Permanent or intensive exposure to MoD can have serious consequences. Appropriate support services have the potential to improve the handling of MoD.

**Objective:**

This article aims to provide an overview of the current state of research on MoD among medical students and young medical professionals in order to sensitize lecturers with responsibility for education and training and doctors in leading positions to the problem.

**Main part:**

This article presents the scientific concept of MoD, known triggers, and options for prevention and intervention. The topic is presented with reference to the changes in patient care in the context of the COVID-19 pandemic and research needs are presented.

**Conclusion:**

The article illustrates the necessity of a German-language, interdisciplinary discourse on MoD among medical students and young professionals.

## Einleitung

Ärzt*innen müssen in ihrer Berufstätigkeit mit einer Vielzahl von Stressoren umgehen, was mit einer erhöhten Gesundheitsgefährdung einhergehen kann. In einer US-amerikanischen Übersichtsarbeit konnte gezeigt werden, dass eine solche Gefährdung nicht nur relevant für Fragen des beruflichen Gesundheitsschutzes ist, sondern auch einen wichtigen Einflussfaktor für Fragen der Sicherheit und der Qualität der Patientenversorgung darstellt [1]. Zudem wurde im Rahmen einer Studie in Deutschland bereits vor Ausbruch der COVID-19-Pandemie eine besondere Gesundheitsgefährdung von Ärzt*innen am Anfang ihrer Berufstätigkeit durch arbeitsbedingte Belastungsfaktoren festgestellt [2]. Die Autor*innen schlussfolgerten, dass eine Veränderung der Rahmenbedingungen notwendig sei, um ein nachhaltig gesundes und effektives Arbeiten zu ermöglichen [2].

Die weltweite COVID-19-Pandemie stellt alle Berufstätigen in der medizinischen Versorgung vor besondere Herausforderungen. Auch in Deutschland bringt sie eine Reihe pandemiebedingter Stressoren für Gesundheitsfachkräfte mit sich [3]. Hierbei sind mit Blick auf berufsethische Anforderungen an Gesundheitspersonal in der Patientenversorgung Aspekte zu nennen, die einen Einfluss auf das professionelle Handlungsvermögen haben können. Fachkräfte in der Gesundheitsversorgung haben die professionelle Verpflichtung übernommen, ihr Handeln an medizinethischen Prinzipien auszurichten. Diese Verantwortungsübernahme beginnt schon in Praxisphasen der Ausbildung. Dazu gehört vor allem: Patient*innen durch ihr Handeln wohlzutun (Beneficence/Wohltun), ihnen nicht zu schaden (Nonmaleficence/Nichtschaden), die Autonomie von Patient*innen zu respektieren (Respekt der Autonomie) und die Versorgungressourcen auf eine gerechte Art und Weise zwischen den Anspruchsgruppen aufzuteilen (Gerechtigkeit; [4]). Dabei sind die Wirkungen der Pandemie und der pandemiebedingten Veränderungen in den medizinischen Versorgungsabläufen nicht für jede Region, jedes Krankenhaus, jedes Behandlungsteam oder Individuum gleich. Sie können mancherorts Veränderungen beinhalten, die es erschweren, die ethischen Werte und Prinzipien zu realisieren. Herausforderungen können zum Beispiel sein:

- Die Versorgung aller Patient*innen muss kontinuierlich und rasch an die aktuelle, sich ständig wandelnde Situation angepasst werden, was vorstrukturierte Handlungsabläufe und Routinen verändert oder unterbricht sowie eine Planbarkeit der Versorgung einschränkt (Wohltun, Nichtschaden und Gerechtigkeit).
- Strikte Sicherheitsmaßnahmen, wie beispielsweise Isolations- und Hygienemaßnahmen, können sich ungünstig auf die Kommunikation zwischen Berufstätigen, Patient*innen und Angehörigen bzw. auf die Zusammenarbeit innerhalb (multiprofessioneller) Teams auswirken (Respekt der Autonomie; [5]).
- Wechselnde Verfahrensanweisungen erfordern eine individuelle und (in Behandlungsteams) eine gemeinsame Ausdeutung in der Umsetzung und schränken mitunter Spielräume zur Anpassung der Behandlung an den Einzelfall ein ([3]; Wohltun, Nichtschaden und Respekt der Autonomie).
- Innerhalb des Gesundheitssystems vollziehen Akteure teilweise einen Perspektivwechsel von einer vorrangig auf das individuelle Patientenwohl gerichteten hin zu einer stark populationszentriert ausgerichteten Versorgung, was von moralischer Verunsicherung bis hin zu moralischen Konflikten führen kann. Dies betrifft Prozesse der Priorisierung medizinischer Maßnahmen, den Umgang mit ressourcenintensiven Therapien, aber auch mit elektiven und präventiven Behandlungsangeboten, also die Verteilung von Ressourcen in Hinblick auf alle Patient*innen, die (intensiv-)medizinische Behandlung in dieser Zeit von drohender Ressourcenknappheit benötigen (Wohltun, Nichtschaden und Gerechtigkeit; [5]).
- Käme es zu einer Ausschöpfung regionaler und überregionaler Ressourcen, dann müssten sich Behandlungsteams und weitere Beteiligte im Krankenhaus der Entscheidung gegen eine indizierte, vom Patienten gewollte Behandlung (Triage) und der damit verbundenen Belastung stellen (Wohltun, Nichtschaden und Gerechtigkeit; [5]).
- Berufstätige im Gesundheitswesen, die Kontakt zu COVID-19-Erkrankten haben, arbeiten unter erhöhter Gefährdung ihrer selbst und ihres persönlichen Umfelds, was nicht nur mit Sorge, mit sozialer Isolation und Stigmatisierung im eigenen Umfeld und im Falle einer eigenen Erkrankung mit weiteren Verunsicherungen einhergehen kann, sondern auch zu einer Selbstdistanzierung im Kontakt mit dem*r Patient*in führen kann. Hierbei sind nicht nur Prinzipien des Wohltuns, Respekt der Autonomie und der Gerechtigkeit berührt, sondern auch Konflikte zwischen der Verpflichtung zur Fürsorge für Patient*innen und der Verpflichtung zur Selbstfürsorge und dem Schutz der Gesundheitsfachkräfte möglich [3, 5].
- Ein Mangel an evidenzbasierten Prognosewerten und Therapieoptionen für Patient*innen mit COVID-19 erfordert Entscheidungen und Behandlung unter Unsicherheit, was wiederum Teamkonflikte im Ringen um adäquate Handlungsstrategien und entsprechend Fragen nach Verantwortlichkeiten und der Verantwortungsübernahme auch für Fehlentscheidungen zur Folge haben könnte.

Diese und weitere pandemiebedingte Herausforderungen können selbst für erfahrenes Gesundheitspersonal belastend sein [6]. Wenig Aufmerksamkeit wird jedoch bisher den zahlreichen Berufseinsteigenden und Studierenden in klinischen Semestern geschenkt, die in den ersten Wochen der COVID-19-Pandemie in Deutschland zur Unterstützung durch ihre Arbeitskraft aufgerufen wurden [7]. Letztere sind noch keine Ärzt*innen, aber übernehmen Aufgaben, die durchaus anspruchsvoll sind und vermutlich vielerorts aufgrund ihres Ad-hoc-Eintritts in die berufliche Tätigkeit zunächst nicht klar definiert wurden. Zusammengefasst werden in diesem Beitrag Medizinstudierende in praktischen Tätigkeiten (bei einer Famulatur, einer Hilfskraftanstellung oder im praktischen Jahr) und approbierte Ärzt*innen am Anfang ihrer Berufstätigkeit (Berufseinsteigende) in den Blick genommen. Die Motivation, zu helfen und die im Studium erworbenen Kompetenzen zur Anwendung zu bringen, ist bei beiden Gruppen vielerorts sehr stark ausgeprägt [8]. Sie können sich jedoch selbst in einer vulnerablen Situation befinden, besonders dann, wenn sie noch wenige Möglichkeiten hatten, Bewältigungsstrategien im Umgang mit moralisch herausfordernden Situationen zu entwickeln. Für eine gemeinsame, aber doch differenzierte Betrachtung dieser beiden Zielgruppen sprechen Bestrebungen der letzten Jahre, Aus- und Weiterbildung mehr miteinander zu verbinden [9].

Es ist davon auszugehen, dass ein erheblicher Teil von Berufseinsteigenden in der Versorgung während der Coronapandemie mit moralischen Konflikten oder pandemiebedingten Einschränkungen der Handlungsmöglichkeiten konfrontiert ist, wobei der Umgang damit zurzeit zu wenig Aufmerksamkeit erfährt. Moralische Konflikte sowie innere oder äußere Einschränkungen der Möglichkeiten, gemäß eigenen moralischen Überzeugungen zu handeln, können mit psychischer Belastung in Form von „moralischem Stress“ (MoS; Englisch: „moral distress“) einhergehen [10–12]. Moralischer Stress kann eine Herausforderung, Bedrohung oder sogar eine Verletzung der beruflichen Integrität bedeuten [13].

MoS ist in der englischsprachigen Literatur ein wissenschaftlich vielbeachtetes Konzept, das in Deutschland bisher kaum bekannt ist. Es gibt einen großen Korpus von englischsprachiger Forschungsliteratur im Kontext der Pflegewissenschaften und -ethik, der konzeptuelle, qualitative und quantitative Forschung umfasst (für einen Überblick: [14–16]). Auch im deutschsprachigen Raum gibt es erste Arbeiten in diesem Bereich [17, 18]. Wir gehen allerdings davon aus, dass die Zugehörigkeit zu einer Berufsgruppe und deren Position in der Hierarchie der Krankenhausversorgung einen wichtigen Aspekt in der Betrachtung des Phänomens darstellen, weshalb die Forschung im Bereich der Pflege nicht ohne Weiteres auf die Forschung zu anderen Berufs- bzw. Akteursgruppen übertragbar erscheint [12].

MoS ist für Berufstätige im Gesundheitswesen in Bezug auf die Entwicklung ihrer Kompetenz im Umgang mit berufsethischen Anforderungen von großer Bedeutung. Eine wissenschaftliche Beschäftigung mit MoS findet vor allem innerhalb der klinischen Ethik statt, innerhalb derer sich Wissenschaftler*innen und Praktiker*innen mit der Umsetzung solcher berufsethischen Anforderungen in der Patientenversorgung im Krankenhaus befassen. Aber das Phänomen kann auch im ambulanten Sektor eine Rolle spielen [19]. Das Konzept MoS wird im Folgenden näher vorgestellt, in Bezug auf die Gruppe der Medizinstudierenden und Berufseinsteigenden in der aktuellen Pandemiesituation analysiert und um eine Beschreibung seitens bestehender Konzepte für einen individuellen und systemischen Umgang mit MoS ergänzt.

## Moralischer Stress (MoS)

### Von der engen Definition von Jameton zur weiten Definition von Fourie

Der Begriff MoS wurde durch den Philosophen Andrew Jameton eingeführt [11]. Für Jameton tritt MoS auf, wenn eine Person eine bestimmte Handlung als richtig ansieht, aber von institutionellen Rahmenbedingungen daran gehindert wird, entsprechend ihrer moralischen Vorstellungen zu handeln. Er differenziert MoS von moralischer Unsicherheit und moralischen Dilemmata. Weitere frühe Autor*innen thematisieren innere Handlungseinschränkungen, wie persönliche Ansprüche und erlebtes Versagen, als zusätzliche Auslöser für MoS [20, 21]. Corley hingegen betont institutionelle Handlungseinschränkungen als Auslöser von MoS, um Institutionen in die Verantwortung zu nehmen, moralisch gebotenes Handeln nicht zu behindern [16]. Der institutionell-kollektive Umgang mit moralischen Herausforderungen wird mittlerweile als „ethisches Arbeitsklima“ (Englisch: „ethical climate“) beforscht [22, 23].

Hanna und Fourie plädieren für eine weiter gefasste Definition von MoS. Von Hanna wird MoS als übergeordnete Kategorie aufgefasst, die in Verbindung mit moralischer Unsicherheit und im Umgang mit moralischen Konflikten auftreten kann [24]. Ähnlich sieht auch Fourie moralischen Stress als Dachbegriff für psychische Reaktionen auf moralische Herausforderungen [10]. Sie nimmt bei ihrer Definition auch andere Berufsgruppen, wie beispielsweise Ärzt*innen, in den Blick, wenn sie folgert: „Moral distress is a psychological response to morally challenging situations such as those of moral constraint or moral conflict, or both“ [10]. Auch Morley et al. beschließen ihre narrative Analyse der konzeptuellen Literatur damit, dass das Vorliegen eines moralischen Ereignisses (auch eines moralischen Konfliktes), die psychische Stressreaktion und die Verbindung von moralischem Ereignis und psychischer Reaktion MoS hinreichend bestimmen würden [25].

Ob nun die enge oder die weite Definition gelten soll, ist Gegenstand aktueller Kontroversen [18]. Unseren Ausführungen liegt die erweiterte, aber gleichzeitig differenzierte Konzeptualisierung von MoS nach Fourie [10, 12] zugrunde (siehe Infobox 1), weil sie in unseren Augen einen guten und klar nachvollziehbaren Mittelweg zwischen Offenheit und Differenziertheit bei der Beschreibung der möglichen (psychischen) Reaktionen auf moralische Herausforderungen darstellt.

Für die Quantifizierung von und den reflektierten Umgang mit MoS ist es wichtig zu definieren, wer MoS erlebt und welche Situationen als Auslöser von MoS bestimmt werden können [12].

### Ursachen und Folgen von MoS bei Medizinstudierenden und ärztlichen Berufseinsteigenden

Medizinstudierende und Berufseinsteigende können eine Reihe von allgemeinen Risikofaktoren für das Erleben von moralischem Stress aufweisen, darunter [15]:

- hohe moralische Sensibilität (bzw. geringe moralische „Abstumpfung“),
- Wissenslücken,
- sich mitverantwortlich fühlen für das Wohl der Patient*innen bei geringer Einbeziehung in die Entscheidung aufgrund hierarchischer Entscheidungsstrukturen.

In einer US-amerikanischen Studie enthielt etwa ein Drittel der Fallbeschreibungen Medizinstudierender Schilderungen von MoS infolge von Handlungseinschränkungen [26]. Trotz ihres hierarchiebedingt geringen Handlungs- und Entscheidungsspielraums identifizierten sich Studierende dennoch mit der Aufgabe der Patientenversorgung [26].

Medizinstudierende erfahren MoS zum Beispiel:

- infolge eines respektlosen Umgangs innerhalb des Teams bzw. im Umgang mit den Patient*innen [27, 28],
- bei Missachtung der Patientenautonomie [29],
- bei Fehlkommunikation [29],
- bei Nichtbehandeln von Patient*innen infolge von Überlastung der Versorgungskapazitäten, von sozialer Ungerechtigkeit oder infolge von Unsicherheit und Überforderung [28, 30],
- bei Übertherapie [28], v. a. im Umgang mit Patient*innen in einer palliativen Situation [31] oder bei geriatrischen Patient*innen [32].

Ist der Auslöser für moralischen Stress zum Beispiel ein Fehlverhalten anderer Mitarbeitender, stellt sich die Frage, wie Personen, die MoS erleben, mit den auslösenden Situationen und ihren Reaktionen darauf umgehen. Allgemein wird in der Stressbewältigungsforschung zwischen einem instrumentellen (problemorientierten) Umgang und einem palliativen (emotionsorientierten) Umgang unterschieden. Während der instrumentelle Umgang auf die Beseitigung oder Lösung der belastenden Problemkonstellation abzielt, ist die palliative Bewältigung auf die direkte Linderung der aversiven Stressemotionen ausgerichtet [33]. Als Hauptgrund, nicht problemorientiert, sondern vorwiegend emotionsorientiert mit MoS umzugehen, gaben Medizinstudierende in einer US-amerikanischen Umfrage beispielsweise ihre untergeordnete Rolle im Team an oder äußerten Sorge darum, einen schlechten Eindruck bei den Vorgesetzten zu hinterlassen [28]. In einem anderen Fall nahmen sie ihr eigenes Handeln als aussichtslos und ihre eigene Meinung als nicht fundiert wahr [32]. Die wichtige Bedeutung, die das Lernen am Modell und ein sogenannter impliziter Lehrplan („hidden curriculum“) für die Sozialisation in der „Arztwerdung“ haben, ist bereits vielfach herausgestellt worden (u. a. [34]). Das Erleben von MoS bedeutet für Medizinstudierende oft ein Lernen am negativen Modell [35], während die Anwesenheit positiver Rollenmodelle zu weniger MoS führen kann [26, 29]. Verschiedene Ursachen konnten bereits nachgewiesen werden, so erleben ärztliche Berufseinsteigende MoS unter anderem:

- durch Übertherapie am Lebensende [36],
- bei wahrgenommener Hilflosigkeit, Leid zu lindern [36],
- bei eingeschränktem Handlungsspielraum durch die gegebenen Hierarchien [36, 37],
- durch Dehumanisierungs- und Rationalisierungsprozesse [36],
- durch ihre eigene Unerfahrenheit und die Hierarchie in Teams [37].

Es ist anzunehmen, dass die anfangs beschriebenen moralischen Herausforderungen bei Medizinstudierenden und ärztlichen Berufseinsteigenden moralischen Stress auslösen können. Es ist beispielsweise denkbar, dass Berufseinsteigende die Erfahrung machen, dass wichtige Behandlungen aufgeschoben werden, um Intensivbetten vorzuhalten, die dann allerdings nicht abgerufen werden. Wenn sich der Zustand des/r Patient*in, der/die die aufgeschobene Behandlung benötigt, zu dem Zeitpunkt der Wiedereinbestellung verschlechtert hat, könnte ein solcher Fall moralischen Stress hervorrufen, v. a. wenn sie direkt involviert sind und Aufgaben in der Behandlung des/der Patient*in oder der Kommunikation mit diesem/er übernommen haben.

Dabei hängt die Wahrnehmung einer moralischen Herausforderung in solchen Situationen bis zu einem gewissen Grad von der persönlichen Erfahrung und den verinnerlichten Wertvorstellungen und Wertehierarchien ab, die das moralische Urteil und die psychische Reaktion prägen können. Welche Art von MoS dann die Folge ist, kann nur anhand des moralischen Urteilsvermögens der Betroffenen und ihres situationsspezifischen Urteils bestimmt werden. Ob z. B. bei der Absage einer wichtigen Behandlung durch den/die Vorgesetzte*n moralische Unsicherheit („Ist das überhaupt moralisch in Ordnung?“) oder ein moralischer Konflikt (z. B. eine Abwägung zwischen der Verpflichtung, einem/er bekannten Patient*in jetzt zu nützen, gegenüber der Verpflichtung, potenziell schwerer betroffenen Patient*innen in der Zukunft zu nützen) besteht oder ob eine Handlungseinschränkung erlebt wird („Jemand verbietet mir, meinem Patienten durch mein Behandlungsangebot zu nützen“), liegt im Auge des Akteurs bzw. auch des Beobachtenden. Unmittelbare Folgen von MoS für Medizinstudierende und Berufseinsteigende können Gefühle von Wut, Verachtung, Scham und Schuld sein [36, 38]. Bei deutschen Medizinstudierenden kann die Wahrnehmung von MoS zu Überlegungen führen, das Studium abzubrechen oder zu wechseln [31]. Analog konnte gezeigt werden, dass MoS bei Berufseinsteigenden mit Burnout korreliert oder zu der Überlegung führen kann, den Beruf oder die Anstellung zu wechseln [39].

### Interventionen für den Umgang mit MoS und Prävention

Für Berufseinsteigende gibt es aktuell unserem Wissen nach keine evidenzbasierten Interventionen zur Verbesserung des Umgangs mit MoS oder zur Prävention seiner negativen (Gesundheits‑)Folgen. Es sind verschiedene Ansätze beschrieben worden, welche zum Teil an anderen Zielgruppen (z. B. Gesundheits- und Krankenpfleger*innen vor und nach Examinierung) bzw. mit anderer Zielsetzung (z. B. Ausbildung, klinische Ethikberatung) erprobt wurden, die das Potenzial haben, sich auf den Umgang mit MoS durch die Zielgruppen dieses Beitrags übertragen zu lassen. Bei so wenigen Anhaltspunkten sind wir daher weit entfernt davon, sagen zu können, ob sich vor dem Hintergrund bisheriger Erkenntnisse in diesem Bereich pandemiespezifische Empfehlungen ableiten lassen.

Vorgestellt werden daher im Folgenden 3 übergreifende Ansätze, die den Umgang mit MoS im Allgemeinen adressieren: A) Hilfe zur Selbsthilfe, B) kollegiale Fallbesprechungen und C) Mentoringprogramme.^1^

#### A) Hilfe zur Selbsthilfe

Am Anfang eines reflektierten Umgangs mit MoS steht seine Identifikation. Um Berufseinsteigende diese (Selbst‑)Erkenntnis zu ermöglichen, ist es notwendig, MoS in der ärztlichen Aus‑, Fort- und Weiterbildung zu thematisieren und mit lebensnahen Beispielen aus der Perspektive ihrer Gruppen zu veranschaulichen [35]. Der nächste Ansatzpunkt kann die aufmerksame Selbstwahrnehmung sein. Dies kann eine Wahrnehmung der eigenen Stärken und Schwächen, die Thematisierung von MoS im Behandlungsteam und den kollegialen Dialog über die auslösende Situation mit anderen Beteiligten bedeuten [40]. Auch die schriftliche Reflexion eigener klinischer Erfahrungen kann zur Identifikation von MoS beitragen [26, 29]. Achtsamkeitstraining hat darüber hinaus das Potenzial, sich positiv auf die Wahrnehmung von MoS auszuwirken [41].

Ein bewährtes Instrument zur Bewältigung von MoS aufgrund von Machtasymmetrien zwischen Pflegenden und beispielsweise Ärzt*innen ist die Handlungsempfehlung „The 4 a’s to rise above moral distress“, die durch ein Gremium der Fachgesellschaft für amerikanische Intensivpflege entwickelt wurde [42]. Die Handlungsempfehlung beinhaltet eine Analyse der Situation, die über die Situation von Pflegenden hinaus hilfreich sein kann. Sie wird in einer modifizierten Form in Infobox 2 dargestellt. Sie beinhaltet eine Art Empowerment-Strategie in 4 Schritten: *A*sk (Fragen), *A*ffirm (Bestätigen), *A*ssess (Einschätzen) und *A*ct (Handeln; [42]). Die einzelnen Schritte sollen wiederholt werden, bis ein Erfolg eintritt [36]. Vergleichbare Ansätze für Medizinstudierende oder Berufseinsteigende sind uns nicht bekannt, aber es wäre z. B. denkbar, eine Adaptation dieser Empowerment-Strategie mit dieser Zielgruppe gemeinsam zu entwickeln.

#### B) Fallbesprechungen in Gruppen

Fallbesprechungen in unterschiedlichen Konstellationen werden seit Langem in der Ausbildung zur klinischen Ethikberatung und im begrenzten Umfang im Medizinstudium eingesetzt (vgl. z. B. [43, 44]). Sie haben das Potenzial, MoS zu reduzieren bzw. die Entstehung von MoS zu verhindern und zu einem positiven ethischen Arbeitsklima beizutragen [45]. Mithilfe der Moral Case Deliberation können unterschiedliche Ziele gleichermaßen verfolgt werden [46]. Es handelt sich dabei um eine retro- oder prospektive ethische Fallbesprechung, die auf einer praktisch-hermeneutischen, dialogischen Ethik basiert und sowohl für homo- als auch heterogene Gruppen geeignet ist. Mitarbeiter*innen des Gesundheitssystems – also auch Berufseinsteigende – können ihre Fallfragen in einen strukturierten Dialog einbringen, der dann von einer/em Ethiker*in mit verschiedenen Konversationsmethoden begleitet wird, um schließlich Antworten auf die moralischen Fragen zu gewinnen und moralische Kompetenzen (weiter) zu entwickeln. Hamric und Epstein haben ein eigenes Modell für die teambasierte Fallberatung bei MoS infolge von Handlungseinschränkungen entwickelt [47], das von klinischen Ethikberater*innen vor Ort als Alternative zur prospektiven klinischen Ethikberatung vor Entscheidungssituationen angeboten wird. Solche Modelle könnten in Angeboten für multiprofessionelle Behandlungsteams realisiert werden.

#### C) Mentoring

Studien konnten zeigen, dass positive Rollenvorbilder einen Einfluss auf den Umgang mit MoS von Medizinstudierenden haben [26, 29]. Für alle Berufseinsteigenden können diese Funktion unmittelbare Vorgesetzte oder erfahrene Kollegen*innen, aber auch designierte Mentor*innen übernehmen, aus deren Erfahrungswissen die Mentees im persönlichen Austausch schöpfen und durch die sie an positiven Rollenvorbildern lernen können [48, 49].

Rosenthal und Clay beschreiben in einer Übersichtsarbeit verschiedene Mentoringprogramme für Medizinstudierende, die sich mit MoS aufgrund von Handlungseinschränkungen im Umgang mit Patient*innen am Lebensende konfrontiert sehen [50]. Ein Ansatz sind Peer-to-Peer-Mentorings für Berufseinsteigende, in denen sich Kolleg*innen gegenseitig unterstützen und beraten. Formelleren Charakter bekommen solche Strategien im Rahmen von Train-the-Trainer-Programmen, in denen die Mentor*innen in moralischer Reflexion und Begleitung geschult werden [50]. Solche Programme könnten um den Aspekt des Umgangs mit MoS erweitert werden.

## Fazit: Forschungs- und Handlungsdesiderat

Das weitgehende Fehlen von Forschung und akademischem Austausch zu MoS in Deutschland ist für uns Autorinnen schwer erklärlich. Erste hiesige Studien zeigen, dass Medizinstudierende in Deutschland schon vor der COVID-19-Pandemie MoS erlebt haben und dass sie durch MoS erhebliche Belastungen erfahren können. So gaben 75 % der 217 Medizinstudierenden einer monozentrischen Fragebogenstudie zu MoS im Rahmen der Versorgung am Lebensende an, mindestens einmal eine der vorgegebenen Situation erlebt zu haben [31]. Ursachen, Umgang und Vorschläge zu verbessertem Umgang mit MoS im praktischen Jahr (PJ) wurden in einer Dissertation unter Betreuung der Erstautorin mittels semistrukturierter Einzelinterviews von 15 Studierenden im praktischen Jahr (PJ) qualitativ analysiert. Dabei zeigen sich vielfältige und komplexe Ursachen für MoS und im Umgang vorwiegend emotionsorientierte Bewältigungsstrategien [38].

Letztlich fehlen allerdings qualitative Studien in Deutschland, die explorieren, welche Formen von pandemiebedingtem moralischen Stress von dieser oder anderen (Berufs‑)Gruppen tatsächlich erlebt werden. Auch das Ausmaß, d. h. zum Beispiel die Häufigkeit und Intensität der Belastung von Berufseinsteigenden, ist bisher kaum erforscht. Folglich ist es schwierig, einen Anstieg im Zuge der COVID-19-Pandemie zu bestimmen. Wir wissen letztlich auch noch nicht, welche Bedeutung MoS im deutschsprachigen Kontext überhaupt haben wird, wenn das Konzept bekannter wird und mehr Forschungsliteratur dazu entsteht. Die moralischen Herausforderungen im Rahmen der COVID-19-Pandemie sind außergewöhnlich, aber sie weisen auch teilweise Faktoren auf, die aus anderen Settings, Berufsgruppen oder Gesundheitssystemen bekannt sind.

Aus unserer Sicht ist die Bedrohung des Gesundheitssystems durch die COVID-19-Pandemie auch ein Anlass, die Stressresistenz und Resilienz des Systems selbst zu überprüfen. Dazu sollten auch die Mitglieder des Systems betrachtet werden und besonders jene, die aufgrund ihrer Arbeitsbedingungen gefährdet sind.

Wir nehmen an, dass MoS bei Medizinstudierenden und Berufseinsteigenden infolge der COVID-19-Pandemie zugenommen hat. Eine Belastung durch MoS könnte andererseits aufgrund der Schaffung von mehr Gesundheitsressourcen in Vorbereitung auf einen rasanten Anstieg von Patient*innen, der dann mancherorts nicht eingetreten ist, auch gesunken sein. Wenn aufgrund einer Überforderung des Gesundheitssystems der Ressourcenmangel und die Allokationszwänge ansteigen, ist eine Zunahme von MoS aufgrund der Notwendigkeit von Priorisierungsentscheidungen zu erwarten. Auf solche Situationen war das deutsche Gesundheitssystem bisher nicht vorbereitet. Das lässt vermuten, dass auch Berufseinsteigende und ihr klinisches Umfeld für diese Situation noch keine ausreichenden Bewältigungsmöglichkeiten für solche Situationen entwickelt haben. Wir halten eine Erforschung der Belastungsintensität durch MoS im Kontext der COVID-19-Pandemie, beispielsweise mittels qualitativer Explorations- und psychometrischer Fragebogenstudien, für notwendig, um weiteren Handlungsbedarf zu bestimmen. Damit einher geht auch die Notwendigkeit, evidenzbasierte Strategien im Umgang mit MoS zu entwickeln. Das macht eine (partizipative) Entwicklung und Wirksamkeitsprüfung von gezielten Interventionen notwendig. Dabei sollten Ansätze zur Selbsthilfe, auf MoS fokussierte Falldiskussionen und (Peer-to-Peer‑)Mentoringprogramme, die den Umgang mit MoS thematisieren (usw.), eine systematische Evaluation erfahren.

Zuletzt sehen wir es auch als notwendig an, verschiedene Berufsgruppen für MoS zu sensibilisieren, einen Diskurs über MoS in Deutschland anzustoßen und zu fördern. Nicht nur Lehrende in Aus‑, Fort- und Weiterbildung von (zukünftigen) Ärzt*innen, sondern auch Personen in Führungspositionen sollen MoS kennen und einen adäquaten Umgang mit MoS fördern. Obligat ist dies nicht nur zum Schutz des Personals vor Gesundheitsschäden, sondern auch als Lehrauftrag und -verantwortlichkeit, um angesichts der moralischen Herausforderungen im Gesundheitswesen nachhaltige, ethisch informierte Praktiken zu etablieren.

### Infobox 1 Unterschiedliche Formen von moralischem Stress. Begriffsbestimmungen nach Fourie [10, 12]

**Moralischer Stress aufgrund von Unsicherheit**

Moralischer Stress aufgrund einer Situation, in der Unsicherheit herrscht, worin das moralische Problem besteht oder welche Prinzipien, Werte oder moralische Pflichten handlungsleitend sein sollen.

*Anhaltspunkt: „Ich weiß nicht, woran ich mich orientieren soll.“*

**Moralischer Stress aufgrund von moralischen Konflikten**

Moralischer Stress aufgrund einer Situation, in der mehrere normative Faktoren (z. B. moralische Prinzipien, Werte oder Pflichten) kollidieren.

**Moralischer Stress aufgrund eines moralischen Dilemmas**

Wie bei moralischen Konflikten, aber verschärft dadurch, dass diese Faktoren miteinander unvereinbare Handlungen erfordern.

*Anhaltspunkt: „Egal was ich tue, ich kann es nicht richtig machen.“*

**Moralischer Stress aufgrund von Einschränkung moralischer Handlungsmöglichkeiten**

Moralischer Stress aufgrund einer Situation, in der eine moralische gebotene

Handlung bekannt ist, aber äußere/innere Barrieren dazu führen, dass diese Handlung nicht ausgeführt wird.

*Anhaltspunkt: „Etwas läuft hier falsch. Das was hier läuft ist nicht das, wofür ich angetreten bin.“*

### Infobox 2 Schrittweise Situationsanalyse bei moralischem Stress. Basierend auf Rushton [42]

1. Was sind in diesem Fall die Ursachen für moralischen Stress?
2. Was steht in diesem Fall auf dem Spiel? (Z. B.: Wer könnte welchen Schaden erleiden?)
3. Welche Handlungsmöglichkeiten gibt es?
4. Was sind die Chancen und Risiken der unterschiedlichen Handlungsmöglichkeiten?
5. Welche Barrieren bestehen, in der Umsetzung der verschiedenen Handlungsmöglichkeiten?
6. Wie würden Sie handeln?
7. Wie hätte die Situation verhindert werden können?
8. Wie könnten zukünftig solche Situationen verhindert werden?

## Footnotes

1 Mit dieser Auswahl erheben wir keinen Anspruch auf Vollständigkeit. Nicht weiter eingegangen wird auf weiterführende Ansätze, die beispielsweise zur Verbesserung des Umgangs mit Stress oder der Konfrontation mit kritischen Ereignissen im Allgemeinen (Selbstfürsorge, Debriefing, Krisenintervention), zur Aus‑, Fort- und Weiterbildung in Medizinethik im Allgemeinen, zum generellen Umgang mit berufs- und organisationsethischen Herausforderungen im klinischen Alltag (klinische Ethikkomitees, Ethikberatung, Leitlinien) oder zur Sozialisation in den Arztberuf abseits von MoS beitragen.

## References

[1] Wallace JE, Lemaire JB, Ghali WA (2009). Physician wellness: a missing quality indicator. Lancet.

[2] Raspe M, Koch P, Zilezinski M (2020). Arbeitsbedingungen und Gesundheitszustand junger Ärzte und professionell Pflegender in deutschen Krankenhäusern (Working conditions and health status of young physicians and nurses in German hospitals). Bundesgesundheitsblatt Gesundheitsforschung Gesundheitsschutz.

[3] Petzold MB, Plag J, Ströhle A (2020). Umgang mit psychischer Belastung bei Gesundheitsfachkräften im Rahmen der Covid-19-Pandemie (Dealing with psychological distress by healthcare professionals during the COVID-19 pandemia). Nervenarzt.

[4] Beauchamp TL, Childress JF (2019). Principles of biomedical ethics.

[5] Morley G, Sese D, Rajendram P, Horsburgh CC (2020) Addressing caregiver moral distress during the COVID-19 pandemic. Cleve Clin J Med. 10.3949/ccjm.87a.ccc047 10.3949/ccjm.87a.ccc047 32518134

[6] Bohlken J, Schömig F, Lemke MR, Pumberger M, Riedel-Heller SG (2020). COVID-19-Pandemie: Belastungen des medizinischen Personals (COVID-19 Pandemic: Stress Experience of Healthcare Workers—A Short Current Review). Psychiatr Prax.

[7] Deutsches Ärzteblatt (Hrsg) (2020) COVID-19: Aufrufe an medizinisches Personal. Deutsches Ärzteblatt vom 19.03.2020. https://www.aerzteblatt.de/nachrichten/111190/COVID-19-Aufrufe-an-medizinisches-Personal. Zugegriffen: 8. Okt. 2020

[8] Pagnin D, de Queiroz V, de Oliveira Filho MA (2013). Burnout and career choice motivation in medical students. Med Teach.

[9] van den Bussche H, Niemann D, Robra B-P (2018). Zuständigkeiten und Konzepte zur ärztlichen Ausbildung und Weiterbildung: Ein Plädoyer für eine Neuorientierung (Responsibilities and concepts for undergraduate and postgraduate medical education in Germany : A plea for a reorientation). Bundesgesundheitsblatt Gesundheitsforschung Gesundheitsschutz.

[10] Fourie C (2015). Moral distress and moral conflict in clinical ethics. Bioethics.

[11] Jameton A (1984). Nursing practice. The ethical issues.

[12] Fourie C (2017). Who is experiencing what kind of moral distress? Distinctions for moving from a narrow to a broad definition of moral distress. AMA J Ethics.

[13] Thomas TA, McCullough LB (2015). A philosophical taxonomy of ethically significant moral distress. J Med Philos.

[14] Schaefer R, Zoboli ELCP, Vieira M (2016). Identification of risk factors for moral distress in nurses: basis for the development of a new assessment tool. Nurs Inq.

[15] Lerkiatbundit S, Borry P (2009). Moral distress part I: critical literature review on definition, magnitude, antecedents and consequences. Thai J Pharm Pract.

[16] Corley MC (2002). Nurse moral distress: a proposed theory and research agenda. Nurs Ethics.

[17] Graeb F (2019). Ethische Konflikte und Moral Distress auf Intensivstationen. Eine quantitative Befragung von Pflegekräften. Best of Pflege.

[18] Monteverde S (2019). Komplexität, Komplizität und moralischer Stress in der Pflege. Ethik Medizin.

[19] Woellert K (2019). Das Klinische Ethikkomitee: Ziele, Strukturen und Aufgaben Klinischer Ethik (The clinical ethic committee: aims, structure, and tasks of clinical ethics). Bundesgesundheitsblatt Gesundheitsforschung Gesundheitsschutz.

[20] Webster GC, Baylis FE, Rubin SB, Zoloth L (2000). Moral residue. Margin of error. The ethics of mistakes in the practice of medicine.

[21] Wilkinson JM (1987). Moral distress in nursing practice: experience and effect. Nurs Forum.

[22] van den Bulcke B, Metaxa V, Reyners AK (2020). Ethical climate and intention to leave among critical care clinicians: an observational study in 68 intensive care units across Europe and the United States. Intensive Care Med.

[23] Koskenvuori J, Numminen O, Suhonen R (2019). Ethical climate in nursing environment: a scoping review. Nurs Ethics.

[24] Hanna DR (2004). Moral distress: the state of the science. Res Theory Nurs Pract.

[25] Morley G, Ives J, Bradbury-Jones C, Irvine F (2019). What is ‘moral distress’? A narrative synthesis of the literature. Nurs Ethics.

[26] Camp M, Sadler J (2019). Moral distress in medical student reflective writing. AJOB Empir Bioeth.

[27] Monrouxe LV, Rees CE, Dennis I, Wells SE (2015). Professionalism dilemmas, moral distress and the healthcare student: insights from two online UK-wide questionnaire studies. BMJ Open.

[28] Wiggleton C, Petrusa E, Loomis K (2010). Medical students’ experiences of moral distress: development of a web-based survey. Acad Med.

[29] Lomis KD, Carpenter RO, Miller BM (2009). Moral distress in the third year of medical school; a descriptive review of student case reflections. Am J Surg.

[30] Schrepel C, Jauregui J, Brown A, Shandro J, Strote J (2019). Navigating cognitive dissonance: a qualitative content analysis exploring medical students’ experiences of moral distress in the emergency department. AEM Educ Train.

[31] Thurn T, Anneser J (2020). Medical students’ experiences of moral distress in end-of-life care. J Palliat Med.

[32] Perni S, Pollack LR, Gonzalez WC, Dzeng E, Baldwin MR (2020). Moral distress and burnout in caring for older adults during medical school training. BMC Med Educ.

[33] Klauer T (2012). Stressbewältigung. Psychotherapeut.

[34] Szauter K, Williams B, Ainsworth MA, Callaway M, Bulik R, Camp MG (2003). Student perceptions of the professional behavior of faculty physicians. Med Educ Online.

[35] Berger JT (2014). Moral distress in medical education and training. J Gen Intern Med.

[36] Dzeng E, Colaianni A, Roland M (2016). Moral distress amongst American physician trainees regarding futile treatments at the end of life: a qualitative study. J Gen Intern Med.

[37] Hilliard R, Harrison C, Madden S (2007). Ethical conflicts and moral distress experienced by paediatric residents during their training. Paediatr Child Health.

[38] Klingler J (2019). Moralischer Stress im Medizinstudium: eine qualitative Interviewstudie mit Studierenden im Praktischen Jahr.

[39] Sajjadi S, Norena M, Wong H, Dodek P (2017). Moral distress and burnout in internal medicine residents. Can Med Educ J.

[40] Hamric AB, Davis WS, Childress MD (2006). Moral distress in health care professionals. Pharos.

[41] Vaclavik EA, Staffileno BA, Carlson E (2018). Moral distress: using mindfulness-based stress reduction interventions to decrease nurse perceptions of distress. Clin J Oncol Nurs.

[42] Rushton CH (2006). Defining and addressing moral distress: tools for critical care nursing leaders. AACN Adv Crit Care.

[43] Neitzke G (2008). Ethik in der medizinischen Aus- und Weiterbildung (Ethics in medical education). Bundesgesundheitsblatt Gesundheitsforschung Gesundheitsschutz.

[44] Steinkamp NL, Gordijn B (2010). Ethik in Klinik und Pflegeeinrichtung. Ein Arbeitsbuch.

[45] Sauerland J, Marotta K, Peinemann MA, Berndt A, Robichaux C (2015). Assessing and addressing moral distress and ethical climate Part II: neonatal and pediatric perspectives. Dimens Crit Care Nurs.

[46] Molewijk AC, Abma T, Stolper M, Widdershoven G (2008). Teaching ethics in the clinic. The theory and practice of moral case deliberation. J Med Ethics.

[47] Hamric AB, Epstein EG (2017). A health system-wide moral distress consultation service: development and evaluation. HEC Forum.

[48] Sambunjak D, Straus SE, Marusic A (2010). A systematic review of qualitative research on the meaning and characteristics of mentoring in academic medicine. J Gen Intern Med.

[49] Bergelt C, Heinen I, Guse J (2018). Mentoring für Studierende in der Medizin: Darstellung und Evaluation eines differenzierten Mentoringprogramms an einer medizinischen Fakultät (Mentoring for medical students : Description and evaluation of a differentiated mentoring program at a medical school). Bundesgesundheitsblatt Gesundheitsforschung Gesundheitsschutz.

[50] Rosenthal MS, Clay M (2017). Initiatives for responding to medical trainees’ moral distress about end-of-life cases. AMA J Ethics.

